# Nutritional and Metabolic Interventions to Prevent and Treat Protein–Energy Wasting in Nondialysis CKD—Narrative Review

**DOI:** 10.3390/nu18030390

**Published:** 2026-01-24

**Authors:** Patrícia Kleinová, Blichová Tímea, Vnučák Matej, Karol Graňák, Kollár Andrej, Ševčíková Katarína, Ivana Dedinská

**Affiliations:** 1 Transplant-Nehrology Department, University Hospital Martin, Kollárova 2, 036 01 Martin, Slovakia; kleinova.pata@gmail.com (P.K.); vnucak.matej@gmail.com (V.M.); granak.k@gmail.com (K.G.); andrej.kollar.01@gmail.com (K.A.); katarina.hudac@gmail.com (Š.K.); ivana.dedinska@uniba.sk (I.D.); 2 Department of Internal Medicine I, Jessenius Medical Faculty, Comenius University, 036 01 Martin, Slovakia

**Keywords:** protein–energy wasting, chronic kidney disease, nutrition

## Abstract

**Background:** Protein–energy wasting (PEW) is a major predictor of morbidity and mortality in patients with chronic kidney disease (CKD), even before the initiation of dialysis. Its multifactorial pathogenesis includes reduced dietary intake, chronic inflammation, metabolic acidosis, hormonal disturbances, and dysbiosis of the gut microbiota. Early recognition and targeted management are crucial for preventing muscle loss, functional decline, and adverse outcomes. **Methods:** This narrative review summarises and integrates current evidence from the literature on nutritional and metabolic interventions to prevent and treat protein–energy wasting in patients with nondialysis chronic kidney disease. Relevant clinical trials, meta-analyses, and experimental studies published up to date were evaluated, focusing on dietary strategies, metabolic modulation, physical exercise, and gut microbiome-targeted therapies. **Results:** Adequate energy and protein intake remain the cornerstone of PEW management, based on available clinical and observational evidence. Individualised diets emphasising high-quality and plant-based proteins, oral nutritional supplements, and ketoanalogues can attenuate muscle wasting. Correction of metabolic acidosis and inflammation enhances protein anabolism and nitrogen balance. Physical exercise acts synergistically with dietary interventions to preserve muscle mass and function. Novel approaches—such as modulating the gut–kidney axis with pre-, pro-, and postbiotics or supplementing with short-chain fatty acids—show promise in improving metabolic and inflammatory profiles. **Conclusions:** The management of PEW in nondialysis CKD requires a personalised approach that integrates nutrition, physical activity, metabolic correction and microbiome modulation. Early, coordinated intervention may help to slow the progression of CKD and improve patient survival and quality of life.

## 1. Introduction

Protein–energy wasting (PEW) is a disease-specific catabolic syndrome characterised by loss of body protein and energy stores (skeletal muscle and adipose tissue), driven by metabolic and inflammatory disturbances associated with chronic illness, particularly chronic kidney disease. This term was introduced in 2009 by the International Society of Renal Nutrition and Metabolism (ISRNM) [1]. Sometimes the PEW is mistakenly confused with malnutrition. Protein–energy malnutrition refers to a state of inadequate intake, absorption, or utilisation of energy and protein. Importantly, PEW may develop despite apparently adequate nutritional intake, reflecting the roles of systemic inflammation, metabolic acidosis, hormonal dysregulation, insulin resistance, and anabolic resistance [1,2]. They are both closely associated with morbidity and mortality in patients with chronic kidney disease. The prevalence of PEW increases proportionally with the stage of chronic kidney disease (CKD). While it is approximately 2% in patients with CKD stage 1–2, it rises to 11–54% in patients with CKD stage 3–5 [1,3]. The problem in clinical practice is the late or no identification of PEW. Early diagnosis of protein–energy wasting is key to planning interventions, as it can be challenging to reverse the consequences of PEW at advanced stages [4]. This review article aims to provide an overview of possible interventions based on the pathophysiological processes underlying PEW, with an emphasis on an individualised approach and plant-dominant low-protein diets (PLADO).

## 2. Diagnosis

The gold standard for diagnosing protein–energy wasting is the 2008 ISRNM criteria, which take into account the four main diagnostic categories shown in Table 1. To confirm PEW, ≥1 criterion from at least three of the four diagnostic categories must be met [5]. However, in clinical practice, this model encounters several problems. The first is the assessment of muscle mass by measuring the mid-arm circumference in the context of PEW dynamics, where the measurer may not always capture the same part of the arm. Another issue is assessing protein and energy intake, which might be significantly affected by the patient’s compliance. For these reasons, the diagnosis of PEW may seem complicated and impractical for clinicians, which ultimately prevents its timely diagnosis with negative implications for the patient’s prognosis. Moreau-Gaudry et al., in an effort to simplify the classification, replaced the criterion of muscle mass reduction with creatinine adjusted for body surface area (sCr/BSA). The advantage of the new criterion in clinical practice is the possibility of earlier diagnosis of protein loss, without the need to wait 3–6 months to demonstrate muscle mass loss [6]. In 2022, Chen et al. proposed a new predictive model, the validity of which was confirmed in 2024 and which has the potential to simplify and replace the current PEW criteria [7,8]. This model includes predictive factors such as body mass index (BMI), gender, albumin, total cholesterol, triacylglycerols, vitamin D, and *N*-terminal pro-B-type natriuretic peptide [8].

## 3. Pathophysiology

**Figure 1:**
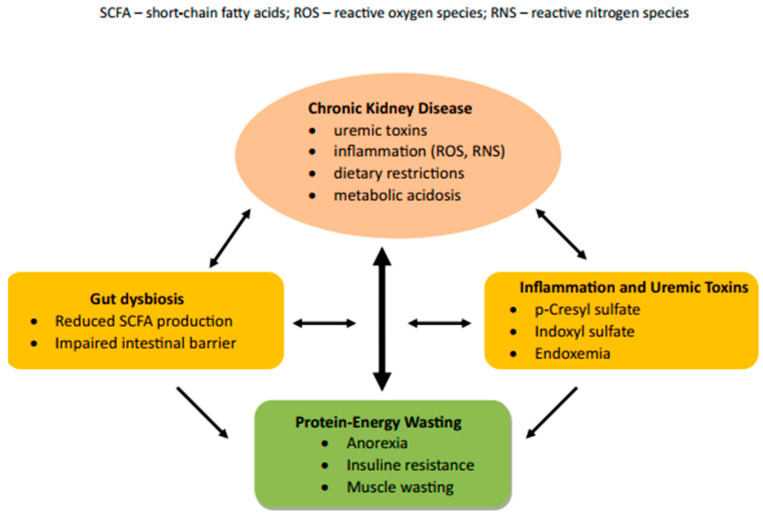
Protein–energy wasting in chronic kidney disease.

Protein–energy wasting in patients with chronic kidney disease arises from a multifactorial process in which individual pathomechanisms interact and potentiate each other (Figure 1). The basis is systemic inflammation, hormonal dysregulation, and an impaired ability to regulate muscle metabolism. A key role is played by reduced food intake (uremic anorexia) in combination with increased energy expenditure at rest. A negative energy balance leads to increased protein utilisation as an alternative energy source, which promotes the formation of uremic toxins and the development of metabolic acidosis (MAC). This complex pathophysiological process, known as anorexia–cachexia syndrome, is the primary driving mechanism of PEW [9].

In patients with advanced CKD, intestinal dysbiosis contributes significantly to the development of PEW, leading to increased production of uremic toxins (*p*-cresyl sulfate, indoxyl sulfate) derived from bacterial fermentation of proteins. At the same time, there is a decrease in bacteria producing short-chain fatty acids (*Faecalibacterium*, *Eubacterium*, *Roseburia*, *Anaerostipes*, *Lactobacillus*) and thus a reduction in short-chain fatty acid (SCFA) levels, which, together with increased intestinal lumen pH, promotes the growth of pathogens (*Enterobacteriaceae*). Dysfunction of the gut–muscle axis alters several metabolic pathways, with a significant impact on the development and progression of PEW [10,11].

The kidneys also play an essential role in maintaining immune homeostasis, which is disrupted in CKD. There is an increase in the concentration of pro-inflammatory cytokines, interleukin 6 (IL-6) and tumour necrosis factor α (TNFα), which contribute to further damage to kidney tissue, creating a vicious circle [12]. Pro-inflammatory cytokines at the central level suppress orexigenic and activate anorexigenic signals, leading to impaired appetite regulation [9]. Their peripheral effects on skeletal muscle, together with MAC, stimulate the ubiquitin–proteasome system, autophagy, and myofibril proteolysis, thereby deepening catabolism [13]. Oxidative stress with increased production of reactive oxygen species (ROS) and nitrogen species (RNS) is another key pathogenetic mechanism. Elevated ROS and RNS levels result from mitochondrial dysfunction, impaired antioxidant processes, reduced adenosine triphosphate production, and activation of signalling pathways that degrade muscle proteins [14].

CKD, like other chronic diseases, is associated with hormonal dysregulation. Despite elevated serum ghrelin levels, patients also tend to exhibit hyperleptinemia due to reduced leptin clearance. Their dysfunction contributes to the pathogenesis of anorexia-cachexia syndrome by impairing anorexigenic neuronal systems, thereby reducing appetite [9,15]. Among non-hemodialed, non-hemodialysed patients, overweight and obesity are associated with lower mortality compared with patients with a BMI < 18.5 kg/m^2^, a phenomenon referred to as the obesity paradox [16]. One possible theory explaining this medical paradox is the hypothesis that excessive nutrition leads to long-term complications, while malnutrition contributes to short-term mortality. The problem, however, is that BMI does not differentiate between lean and fat mass, and in patients with CKD, it may be overestimated in the presence of oedema. The best tool for accurately determining body composition is bioimpedance. A study conducted in non-dialysis patients found that higher muscle mass is associated with lower mortality and fewer cardiovascular events [17,18]. Elevated glucocorticoid levels, to which MAC also contributes, promote proteolysis, inhibit the mTOR pathway, and reduce the synthesis of insulin-like growth factor-1 (IGF-1), thereby disrupting anabolic signals in muscles. Sex hormones also play an essential role—low testosterone in men and reduced oestrogen levels in women impair muscle strength and regeneration [10]. Insulin resistance, which appears early in CKD, results from an increased leptin/adiponectin ratio and persistent inflammation. It leads to reduced mTOR pathway activation and, subsequently, decreased protein synthesis, both of which significantly contribute to the development of PEW [14].

Hypovitaminosis D is a common complication of CKD. Although the exact mechanism of its effect on muscle mass is not fully understood, vitamin D deficiency is thought to be associated with impaired myogenesis in patients with CKD [10].

## 4. Intervention

**Figure 2:**
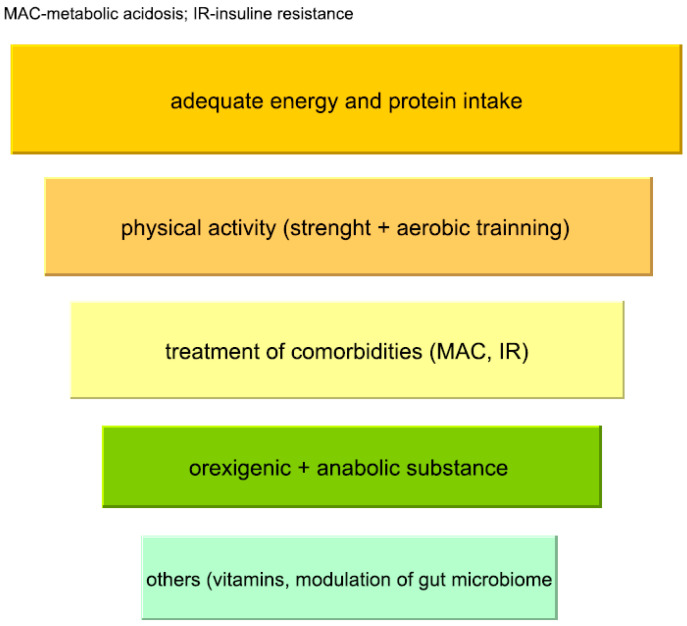
Interventional approaches of prevention and treatment of protein–energy wasting.

PEW represents a dynamic “spiral” of interrelated factors that influence each other, and its prevention and management require a multi-level approach. Strategies should be individualised and optimised to meet each patient’s specific needs. While some therapeutic approaches are supported by more robust evidence, others remain exploratory and await further validation and incorporation into clinical recommendations. An overview of the main intervention domains discussed in this review is presented in Figure 2. Given the heterogeneity of available data across individual interventions, the strength and limitations of the evidence are summarised in Table 2.

### 4.1. Nutritional Intervention in Prevention of PEW

In patients with CKD who do not require haemodialysis, a neutral to slightly positive nitrogen balance, corresponding to an energy intake of 30–35 kcal/kg body weight/day, is necessary to prevent the development of PEW. When prescribing daily energy intake, factors that increase caloric requirements, such as younger age, male gender, hyperparathyroidism, hyperglycaemia, or acute illness, should be considered [52].

In patients with CKD stages 3–5, low-protein diets have been widely studied as a strategy to reduce intraglomerular pressure and metabolic burden. Available evidence suggests that, when combined with adequate energy intake, low-protein diets may be associated with slower CKD progression in selected patients, although results remain heterogeneous [53]. However, nutritional guidelines on the recommended daily protein intake differ between the two leading organisations—Kidney Disease: Improving Global Outcomes (KDIGO) and Kidney Disease Outcomes Quality Initiative (KDOQI) (Table 3) [54].

In selected patients without active PEW, a very low-protein diet (VLPD, 0.3–0.4 g/kg/day) may also be indicated under strict nutritional supervision, except in patients with diabetic nephropathy or nephrotic syndrome. VLPD must always be supplemented with essential amino acids or their ketoanalogues to prevent dietary deficiencies. According to KDIGO, ketoanalogues are only indicated as part of VLPD, but their use in low-protein diets (LPD) is not contraindicated. Supplementation replenishes deaminated essential amino acids, which are converted to branched-chain amino acids in the urea cycle without the formation of nitrogen [55].

The combination of ketoanalogues with a low-protein diet has been shown to be feasible and well tolerated, without adverse effects on renal function, in both CKD and post-transplant populations (Kleinová et al.) [56]. However, the question remains whether LPD can contribute negatively to PEW. In patients with a typical “Western diet,” daily protein and phosphorus intake is often excessive. According to the KDIGO 2024, adequate energy intake allows flexibility in protein prescription, with emphasis on protein quality and bioavailability rather than quantity alone [19]. High-bioavailability protein sources have therefore been proposed as a strategy to maintain essential amino acid intake when total protein intake is reduced [20]. The bioavailability of protein is assessed using the Digestible Indispensable Amino Acid Score (DIAAS), defined by the Food and Agriculture Organization of the United Nations (FAO) and World Health Organization (WHO) in 2011. DIAAS assesses the actual digestible amount of essential amino acids (EA) in the distal ileum relative to a reference protein. An overview of foods with high protein bioavailability is provided in Table 4 [57,58,59].

The KDIGO 2024 Clinical Practice Guideline emphasises healthy dietary patterns with a higher proportion of plant-based foods and minimisation of ultra-processed foods (e.g., sausages, sweets, sweetened beverages) in patients with CKD [19]. An adequately designed plant-based diet may meet nutritional requirements in patients with CKD; however, this requires careful dietary planning, regular monitoring, and close collaboration between the patient, nephrologist, and renal dietitian, as confirmed by studies in patients with CKD [60].

As kidney function declines, reduced renal phosphate excretion promotes phosphate retention and maladaptive endocrine responses, including elevated fibroblast growth factor 23 levels and reduced calcitriol synthesis [61]. Phosphate retention suppresses renal 1α-hydroxylase activation, leading to decreased 1,25(OH)_2_D levels, which may further impair muscle function and have been associated with an increased risk of sarcopenia in patients with CKD [62]. Hyperphosphatemia has also been linked to pro-inflammatory responses, potentially exacerbating the chronic inflammatory milieu that drives anorexia–cachexia syndrome and anabolic resistance [63]. In addition, excessive dietary phosphate intake may disrupt gut microbiota composition and intestinal barrier integrity, as suggested by experimental studies, thereby amplifying endotoxemia and systemic inflammation [64]. Experimental and translational studies suggest that high phosphate exposure can directly contribute to skeletal muscle wasting and impaired myogenic differentiation, whereas phosphate restriction may ameliorate muscle wasting in experimental CKD models [65]. One potential advantage of plant-dominant dietary patterns in chronic kidney disease is the lower intestinal bioavailability of dietary phosphate, as phosphorus in plant foods is predominantly bound to phytates, which are poorly digested by humans. By reducing intestinal phosphate absorption, this dietary pattern may help attenuate phosphate toxicity and its downstream metabolic and inflammatory effects in CKD [20,61,66]. In support of this concept, a study by Moorthi et al. demonstrated that a diet providing 70% of protein from plant sources significantly reduced urinary phosphorus excretion in patients with CKD [67].

Although plant-based diets often contain higher levels of potassium, available evidence suggests that, with appropriate dietary planning and monitoring, they are not consistently associated with a higher risk of hyperkalaemia. Patients with CKD who take angiotensin-converting enzyme inhibitors or angiotensin receptor blockers (approximately 87% of patients), as well as those with advanced CKD (eGFR < 30 mL/min/1.73 m^2^) and reduced tubular potassium excretion, are at increased risk of hyperkalaemia. In these patients, careful selection of plant-based foods, portion control, and regular biochemical monitoring are key to maintaining serum potassium levels within the range of 3.5–5.5 mmol/L [68]. Nowadays, tables showing the exact amount of potassium in a given piece of fruit or vegetable are readily available (on websites and in brochures), which can be particularly beneficial for patients with CKD in determining how much they can eat to maintain normokalaemia. Vegetables and fruits, particularly in the early stages of CKD, are rich in dietary fibre, which promotes regular bowel movements and may help maintain potassium homeostasis while favourably modulating the gut microbiome [69]. Plant-based diets have an alkalizing effect due to organic anions (e.g., malate, citrate), which reduce the dietary acid load [67]. Carbohydrate intake from fruits may facilitate intracellular potassium uptake, contributing to short-term potassium distribution [56]. Fibre encourages the growth of SCFA-producing bacteria, improving epithelial barrier integrity and reducing inflammation associated with CKD progression. In contrast, animal proteins promote proteolytic microbes that produce uremic toxins, which negatively affect disease progression [67].

Dietary patterns characterised by a higher proportion of plant-based proteins have been associated with a more favourable cardiometabolic risk profile in patients with CKD. Extensive prospective cohort studies have shown that a higher dietary plant-to-animal protein ratio is associated with a lower risk of cardiovascular disease and coronary heart disease [70]. Proposed mechanisms include favourable changes in lipid profile (lower low-density lipoprotein and total cholesterol levels), reduced oxidative stress and inflammation, lower dietary acid and phosphate load, improved endothelial function, and reduced trimethylamine N-oxide production [55,70,71,72,73,74,75,76]. A comprehensive comparison of plant and animal proteins in the context of chronic kidney disease is shown in Table 5.

Micronutrient deficiencies, including iron, zinc, vitamin D, and vitamin B_12_, may occur in patients adhering to low-protein plant-based dietary patterns. The bioavailability of some micronutrients may be lower in predominantly plant-based diets, and chronic kidney disease itself further increases the risk of deficiency. Micronutrient deficiencies may contribute to the development or progression of protein–energy wasting. Accordingly, KDIGO recommendations emphasise an individualised approach with regular monitoring of micronutrient status and targeted supplementation when indicated [19,78].

Overall, plant-dominant low-protein diets should be viewed not as a universal solution but as a potentially beneficial dietary pattern for selected patients, requiring individualisation based on CKD stage, metabolic stability, comorbidities, and patient adherence.

### 4.2. Nutritional Intervention in Metabolically Unstable Patients with Active PEW

In metabolically unstable patients with active protein–energy wasting or risk factors for PEW (e.g., inflammatory disease), very low-protein diets should be avoided, as reflected in current KDIGO guidance [54]. In such situations, increased energy intake and higher protein provision (approximately 1.0–1.2 g/kg body weight/day) are commonly recommended to support anabolism and prevent further nutritional deterioration [52]. According to the European Society for Clinical Nutrition and Metabolism (ESPEN), medical nutritional therapy should be considered in patients with AKI, AKI on CKD, and CKD, and may include oral nutritional supplements, enteral nutrition (EN), or parenteral nutrition (PN) depending on clinical status [21]. Even patients with the above-mentioned kidney impairment who are hospitalised for more than 48 h in an intensive care unit should be provided with nutritional support to prevent malnutrition [21].

Oral nutritional supplements (ONSs) are commonly used as an initial nutritional intervention when oral intake is insufficient. Today, various forms of ONSs are available on the market—drinks, puddings, powders—tailored to the specific needs of patients with chronic kidney disease (renal-specific ONS), for example, lower electrolyte and fluid content, as well as taste preferences [52]. ONSs should be taken two to three times a day, preferably about 1 h after meals, to achieve nutritional goals more easily. In hospitalised populations with comorbidities, nutritional supplementation has been associated with improvements in nutritional status and reductions in complications and unplanned rehospitalizations; effects on mortality appear variable and depend on the patient population and study design. Based on available evidence, similar benefits may extend to polymorbid patients with AKI or CKD, although data specific to nondialysis CKD remain limited [21]. When evaluating the effectiveness of ONS, it is essential to monitor not only nutritional status but also compliance and tolerability, with a focus on gastrointestinal symptoms [52]. Some studies have shown high rates of non-compliance and intolerance with long-term use of ONS [22]. Data on ONS use in nondialysis CKD are limited; however, studies in dialysis populations have reported improvements in serum albumin, body mass index, and inflammatory markers, without significant increases in serum phosphate or potassium concentrations [23,24,25].

In patients who are unable to meet approximately 70% of their daily macronutrient requirements by oral intake, escalation to enteral nutrition, parenteral nutrition, or a combination of both should be considered [21]. A meta-analysis of 27 studies comparing a control group with patients receiving enteral nutrition reported increased protein intake and positive effects on patient weight [26]. Total parenteral nutrition (TPN) may be used to meet nutritional requirements in patients who cannot tolerate oral or enteral intake and is typically reserved for short-term use in acute clinical settings, provided that appropriate monitoring is ensured [52]. TPN in patients with CKD is a specific topic, and the available literature is limited. The nutritional requirements of acutely ill patients with CKD are generally considered comparable to those of patients with acute kidney injury who do not require renal replacement therapy. Initial TPN is commonly initiated at approximately 50% of estimated requirements and administered gradually to minimise the risk of metabolic complications. Table 6 lists the recommended parenteral nutrient intake for patients with acute kidney injury (AKI) or CKD who do not require renal replacement therapy [27].

Specially modified amino acid solutions formulated for patients with renal failure have been associated with improved nitrogen balance and metabolic tolerance in selected clinical settings. In contrast, solutions containing only essential amino acids have not demonstrated clear clinical advantages, and their routine use is currently not supported by available evidence. Lipid emulsions as a source of energy should be administered with caution due to reduced hydrolysis and elimination in AKI and CKD, and plasma triacylglycerol concentrations should be monitored. Carbohydrates are typically provided as glucose, and during initiation of TPN, blood glucose levels often require adjustment with insulin to maintain normoglycaemia, given the high prevalence of insulin resistance. Water-soluble vitamins are often supplemented at higher-than-standard doses in patients receiving parenteral nutrition, including vitamin C, for which excessive dosing (≥200 mg/day) should be avoided due to the risk of secondary oxalosis. Of the fat-soluble vitamins, vitamin D supplementation is essential; in acutely ill patients with CKD or AKI, vitamin A and E supplementation is also necessary. Electrolyte intake is determined individually, based on laboratory results and the patient’s clinical condition [28].

However, the question remains as to when to start administering medical nutrition. Studies in hospitalised patients with multiple comorbidities suggest potential benefits of early nutritional support initiated within 48 h of admission [21]. On the other hand, a study by Gunst et al. showed that patients with stage 2 AKI who received early parenteral nutrition experienced delayed recovery [28]. Similarly, in a meta-analysis of 18 studies (3347 patients) comparing enteral and parenteral nutrition, a higher incidence of infectious complications was observed in patients receiving PN, but this was associated with higher calorie intake [29]. According to ESPEN recommendations, in the case of ONS and EN contraindications, PN administration should be started within three to seven days. To minimise the risk of refeeding syndrome, PN should be initiated at reduced doses and advanced gradually [21].

### 4.3. Exercise Intervention

The combination of nutritional intervention and physical activity, particularly resistance training, has a strong physiological rationale and is considered a promising strategy to support anabolic processes in patients with protein–energy wasting. Both strength and aerobic training increase muscle perfusion and the activity of amino acid transporters, thereby promoting the entry of amino acids into muscle cells. Experimental and clinical studies have demonstrated that resistance exercise acutely increases myofibrillar protein synthesis, with effects persisting for approximately 24–48 h (“anabolic window”) [30]. During this period, adequate amino acid availability appears important to maximise the anabolic response to exercise. The available literature does not provide precise data on the preferred protein source; emphasis is mainly on adequate leucine content. In addition to its role as a substrate for protein synthesis, leucine also acts as a signalling activator of the mTOR anabolic pathway, which, when activated, inhibits proteolysis [31]. An interesting finding is that load size does not significantly affect the increase in muscle fibres [30]. Aerobic physical activity reduces insulin resistance, improves adipocyte function, lowers markers of oxidative stress, and benefits the lipid profile [32,33].

A meta-analysis including 10 randomised controlled trials (334 patients) evaluating resistance training in patients with chronic kidney disease reported improvements in several parameters of physical and metabolic health. Reported benefits included improvements in physical function (notably grip strength), reductions in inflammatory markers (e.g., C-reactive protein), favourable metabolic changes (increases in lean body mass and reductions in glycated haemoglobin), and improved cardiopulmonary performance, such as increased six-minute walk distance [34]. Although the synergistic effect of exercise and nutritional intervention has a clear theoretical basis at the metabolic level, randomised controlled trials to date have not demonstrated a significant impact of these combined interventions on BMI, lean body mass, muscle strength, or quality of life. However, these studies included relatively few patients and showed significant heterogeneity [35].

Although the direct effect of strength training on specific parameters has not been clearly demonstrated in some studies, the inclusion of aerobic physical activity in the daily routine of patients with CKD in the pre-dialysis stage appears beneficial and supported by available evidence.

### 4.4. Hormonal and Metabolic Intervention

Hormonal disturbances are among the contributing factors to protein–energy wasting, and their modulation has therefore been explored as a potential therapeutic strategy. According to available data, hormonal interventions such as growth hormone or anabolic steroids, administered alone or in combination with nutritional support, have been associated with improvements in nitrogen balance in selected patient populations and have therefore been explored as potential adjunctive approaches in the management of PEW [50]. Administration of recombinant human insulin-like growth factor-1 has been reported to improve nitrogen balance and alter mineral metabolism, including reductions in serum phosphorus and increases in calcium levels, in patients undergoing peritoneal dialysis [51]. However, on the other hand, hormone therapy is associated with adverse effects such as fluid retention, glucose metabolism disorders, cardiovascular risk, androgenic side effects, and theoretical neoplastic risk. However, only a limited number of studies in the available literature have investigated the use of anabolic hormones in the treatment of PEW. Notably, robust data in nondialysis CKD patients with PEW are lacking, and such interventions should be carefully considered experimental and adjunctive.

Metabolic acidosis, as one of the complications of CKD, contributes to the development and progression of PEW. Several studies suggest that correction of metabolic acidosis with sodium bicarbonate may be associated with slower decline in kidney function and improvements in selected metabolic and nutritional parameters, although results remain heterogeneous [36]. Observational studies have reported increased mortality at both low (<17 mmol/L) and high (>27 mmol/L) serum bicarbonate concentrations. According to the 2024 KDIGO guideline, treatment of metabolic acidosis should be considered when serum bicarbonate levels fall below 17 mmol/L. Bicarbonate supplementation in patients with CKD stages 3–5 is generally aimed at maintaining serum bicarbonate within the normal range, while avoiding both persistent acidosis and overt alkalosis [16]. In a study comparing dietary alkalization (fruits and vegetables) and bicarbonate supplementation, both groups showed improvements in renal function and markers of damage, with no significant differences between groups. Among other findings, patients following a prescribed fruit- and vegetable-based dietary intervention demonstrated increased urinary potassium excretion without concomitant increases in serum potassium concentrations under controlled study conditions [37]. In a study by Dubey et al., bicarbonate supplementation was independently associated with more favourable body composition parameters, including mid-arm muscle circumference and lean body mass [38]. However, during bicarbonate supplementation, we must be cautious due to potential risks of sodium load, such as volume overload and elevated blood pressure, particularly in patients with cardiovascular comorbidities. Therapy should be accompanied by regular clinical and biochemical monitoring. Correction of metabolic acidosis should go hand in hand with nutritional support, as it is a relatively inexpensive and straightforward adjunctive strategy that may help mitigate metabolic contributors to PEW in patients with chronic kidney disease.

### 4.5. Gut Microbiome Intervention

Diet, exercise, and nutraceuticals, including prebiotics, probiotics, synbiotics, and postbiotics, can influence the gut microbiome. However, according to the available literature, no studies have directly focused on modulating the gut microbiome in patients with CKD in the context of PEW-related changes. The evaluation of markers of muscle loss has been primarily conducted in animal models [39]. To date, there are no clinical studies in patients with CKD whose primary goal is to improve PEW through microbiota modification; therefore, the available evidence is derived predominantly from studies conducted in CKD patients without targeted PEW interventions.

According to the International Scientific Association for Probiotics and Prebiotics, prebiotics are substrates selectively utilised by host microorganisms that confer a health benefit to the host [40]. Common prebiotics found in whole grains, fruits, and vegetables are inulin and fructooligosaccharides. A small study in animal models reported a decrease in uremic toxins when oligofructose enriched with inulin was supplemented to rats with CKD [41].

Probiotics are active microorganisms that colonise the human gastrointestinal tract and help maintain microbiological balance. Animal models have shown that probiotics containing Lactobacillus slow the progression of CKD [42]. In a 2023 study, administration of probiotics containing Lactobacillus and Bifidobacterium was associated with reductions in inflammatory markers, including tumour necrosis factor-α and interleukin-6, which are commonly elevated in CKD and PEW [43]. Reductions in inflammatory markers and uremic toxins have also been reported in patients adhering to a low-protein diet [44].

Synbiotics are combinations of live microorganisms and substrates selectively utilised by host microorganisms that provide health benefits [45]. Synbiotic therapy led to reductions in indoxyl sulfate and *p*-cresyl sulfate concentrations over 6 weeks of administration in patients with CKD without dialysis [45].

ISAPP defines postbiotics as natural metabolites produced by microorganisms that have a positive biological effect on the host but are not alive [46]. Key postbiotics include SCFAs—butyrate, propionate, and acetate—which are produced by intestinal bacteria fermenting fibre. Reduced SCFA production in patients with CKD has been associated with increased endotoxemia and markers of muscle catabolism, potentially contributing to the development of PEW. Due to their anti-inflammatory, metabolic, and immunity-modulating effects, they can potentially influence PEW [39].

All of the above-mentioned biotic products can be supported through an adequate diet. As a modulator of the gut microbiota, dietary patterns rich in fermentable fibre may promote the growth of beneficial bacteria and increase SCFA production. Plant-based dietary patterns provide higher amounts of fermentable substrates and bioactive compounds, which have been associated with improved intestinal barrier function, lower systemic inflammation, and reduced generation of certain uremic toxins [47,48,49].

To date, evidence remains insufficient to support routine use of biotic interventions in the prevention or treatment of PEW; therefore, their application should be individualised and, where possible, guided by documentation of intestinal dysbiosis.

### 4.6. Others

Anorexia associated with protein–energy wasting has prompted interest in the use of pharmacological appetite stimulants; however, these agents have not been systematically studied in patients with PEW and are primarily used in other catabolic conditions. These substances include megestrol acetate, dronabinol, cyproheptadine, melatonin, thalidomide, and ghrelin. Anorexia associated with protein–energy wasting has prompted interest in the use of pharmacological appetite stimulants; however, these agents have not been systematically studied in patients with PEW and are primarily used in other catabolic conditions. Megestrol acetate has been shown to exert orexigenic effects, potentially mediated by reductions in interleukin-6 and tumour necrosis factor-α, and is associated with increases in body weight, predominantly due to gains in adipose tissue. The disadvantage of its use is the risk of adrenal suppression, hypogonadism with decreased libido, fluid retention with oedema, atrial hypertension and an increased risk of thromboembolism, which limit its use in patients with CKD [50].

Although pharmacological appetite stimulants have been explored as potential adjunctive options in the management of anorexia associated with PEW, their use in patients with CKD remains limited due to the lack of robust clinical evidence. The available evidence is predominantly short-term, often derived from small cohorts, and does not allow for an assessment of the long-term efficacy or safety of these drugs. For these reasons, any consideration of appetite stimulants should be highly selective, based on careful evaluation of potential risks and benefits, and ideally embedded within a multidisciplinary management strategy combining nutritional intervention, physical activity, and optimisation of comorbid conditions.

Such a comprehensive approach remains key to the effective PEW prevention and treatment, while pharmacological stimulants should be considered more as a supportive rather than a first-line therapy.

## 5. Conclusions

Protein–energy wasting is a complex and clinically significant complication in patients with chronic kidney disease that requires early recognition, particularly in nondialysis populations, in whom its manifestations may be less apparent. Individualised nutritional intervention represents the cornerstone of both prevention and management of PEW.

In metabolically stable patients with CKD, current clinical guidance supports an adequate energy intake (approximately 30–35 kcal/kg/day) and a low-protein dietary approach with a preference for plant-based protein sources, while very low-protein diets should be reserved for carefully selected patients without active PEW and implemented only with appropriate ketoanalogue supplementation. In contrast, in the presence of metabolic instability or established PEW, higher protein provision (approximately 1.0–1.2 g/kg/day) is generally required, and oral nutritional supplementation may be considered. When oral intake remains insufficient, escalation to enteral or parenteral nutrition should be evaluated on an individual basis.

The effectiveness of nutritional therapy appears greatest when integrated with regular physical activity, particularly resistance exercise. Modulation of the gut microbiome represents a promising but still exploratory area; dietary strategies rich in fermentable fibre remain the primary approach, while the use of nutraceutical supplements may be considered selectively. Correction of metabolic derangements, especially metabolic acidosis, constitutes an important adjunctive strategy. In contrast, the role of hormonal or orexigenic therapies remains uncertain and should be regarded as marginal and experimental rather than routine.

Optimal management of PEW therefore requires a multidisciplinary approach with systematic monitoring of nutritional status. However, the evidence base supporting several interventions—particularly the use of oral nutritional supplements in nondialysis CKD—remains limited, restricting the strength of formal recommendations. A pragmatic strategy to improve future implementation includes routine nutritional risk screening in outpatient nephrology practice and early engagement of patients with low energy or protein intake in structured dietary interventions.

## Figures and Tables

**Table 1:** Diagnosis criteria of protein energy wasting [5].

Category	Parameter	Cut-Off
*Biochemical* *parameters*	Serum albumin	<3.8 g/dL
	Serum prealbumin (transthyretin)	<30 mg/dL
	Serum cholesterol	<100 mg/dL
*Body mass*	BMI	<23 kg/m^2^
	Unintentional weight loss	5% over 3 months or 10% over 6 months
	Total body fat percentage	<10%
*Muscle mass*	Muscle wasting	5% over 3 months or 10% over 6 months
	Mid-arm muscle circumference	>10% reduction vs. 50th percentile
*Dietary intake*	Low DPI	<0.8 g/kg/day for ≥2 months
	Low energy intake	<25 kcal/kg/day for ≥2 months

BMI—body mass index; DPI—daily protein intake.

**Table 2:** Evidence summary of interventions targeting PEW in CKD.

Intervention	Study Type	CKD Stage/Population	Key Evidence Summary	Main Limitations
*Low-protein diet (LPD)* [19,20]	Observational studies, small RCTs	CKD stages 3–5, nondialysis	Reduced uremic and phosphate load; neutral nutritional status if energy intake is adequate	Heterogeneous designs; limited PEW-specific outcomes
*Very low-protein diet (VLPD) + ketoanalogues* [19,20]	Small RCTs, observational cohorts	CKD stages 4–5, metabolically stable	Maintains nitrogen balance while allowing protein restriction	Not suitable in PEW or metabolic instability; limited long-term data
*Oral nutritional supplements (ONS)* [21,22,23,24,25]	RCTs, observational studies	Dialysis and nondialysis CKD; hospitalised patients	Improved energy/protein intake; dialysis studies show increased albumin and BMI	Limited data in nondialysis CKD; adherence issues
*Enteral nutrition (EN)* [21,26]	Observational studies, meta-analyses	AKI, CKD, ICU	Improves protein intake and body weight when oral intake is insufficient	Limited CKD-specific trials; tolerance issues
*Parenteral nutrition (PN)* [21,27,28,29]	Observational studies, RCTs	AKI and CKD without RRT; ICU	Ensures nutritional support when oral/EN is not feasible	Infection risk, metabolic complications, sparse CKD data
*Exercise (resistance ± aerobic)* [30,31,32,33,34,35]	RCTs, meta-analyses	CKD stages 3–5, dialysis	Improves physical function, inflammation, and metabolic markers	Small cohorts; heterogeneous protocols; limited PEW endpoints
*Correction of metabolic acidosis* [16,36,37,38]	RCTs, observational studies	CKD stages 3–5	Associated with improved metabolic parameters and body composition	Sodium load; target bicarbonate levels debated
*Microbiome interventions* [39,40,41,42,43,44,45,46,47,48,49]	Animal studies; small human trials	CKD, mostly without PEW targeting	Reduced uremic toxins; improved inflammatory markers	Very limited PEW-specific clinical data
*Hormonal/orexigenic therapy* [50,51]	Small trials, observational studies	Mostly dialysis populations	Short-term increase in appetite or nitrogen balance	Safety concerns; lack of nondialysis CKD data

AKI—acute kidney injury, BMI—body mass index, CKD—chronic kidney disease, EN—enteral nutrition, ICU—intensive care unit, LPD—low-protein diet, ONS—oral nutritional supplements, PEW—protein–energy wasting, PN—parenteral nutrition, RCT—randomised controlled trial, RRT—renal replacement therapy, VLPD—very low-protein diet.

**Table 3:** Recommendations for daily protein intake for non-dialysis chronic kidney disease patients [19,52].

	KDIGO (2024) [19]	KDOQI (2020) [52]
*Energy (kcal/kg of body weight/day)*	not specified	30–35
*LPD (g/kg of body weight/day)*	0.6–0.8	0.55–0.6
*VLPD (g/kg of body weight/day)* *	0.3–0.4 + EA or KA	0.28–0.43 + EA or KA

KDIGO—Kidney Disease: Improving Global Outcomes, KDOQI—Kidney Disease Outcomes Quality Initiative, LPD—low protein diet, VLPD—very low protein diet, EA—essential amino acids, KA—ketoanalogues. * metabolic stable patient without diabetes mellitus or nephrotic syndrome.

**Table 4:** High biological value protein sources and their relevance in CKD [57,58,59].

Food Item	Protein (g/100 g) ^1^	DIAAS ^2^	Biological Value ^3^	Clinical Note for CKD ^4^
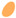 *Whole egg*	12.5	1.13	100	Reference protein; high quality, suitable in limited amounts
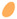 *Egg white*	11	1.14	100	Highest quality; very low phosphorus content—ideal in CKD stages 3–5
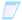 *Milk (low-fat)*	3.4	1.00	91	Good quality; moderate use recommended, monitor P and K intake
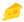 *Cottage cheese*	11–13	1.05	90–95	High-quality protein; consider calcium and phosphorus content
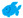 *Fish (cod, salmon)*	18–22	1.00	90–100	High-quality, easily digestible source of omega-3 fatty acids
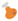 *Chicken*	21	0.90–0.95	80–90	Good quality; low saturated fat content
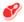 *Beef*	20–22	~0.92	80–90	High phosphorus levels—monitor in CKD
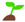 *Tofu (soy protein)*	8–10	0.90	~90	Best plant protein; suitable for a low-protein diet
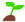 *Tempeh*	18–20	~0.95	~90	Fermented soy product, easy to digest
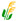 *Rice + lentils (combined)*	variable	0.75–0.85	70–80	Complementary amino acids (methionine + lysine); improved plant protein quality

CKD—chronic kidney disease. ^1^ Source: USDA Food Data Central (2024) [59]. ^2^ DIAAS: Digestible Indispensable Amino Acid Score (FAO/WHO, 2013) [57]. ^3^ BV: Biological Value—proportion of absorbed protein utilised for body protein synthesis [57]. ^4^ Based on KDOQI Clinical Practice Guideline for Nutrition in CKD (2020) [52].

**Table 5:** Comparison of plants and Animal Proteins in chronic kidney disease [55,70,71,72,73,74,75,76,77].

Parameter	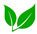 Plant-Based Proteins	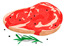 Animal-Based Proteins
*Biological Value/DIAAS*	Lower DIAAS, but adequate if mixed sources are consumed	Higher DIAAS, complete essential amino acids
*Phosphorus Bioavailability*	Lower (phytate-bound, ~30–40% absorbed)	High (~70–80% absorbed)
*Acid Load*	Alkalinizing effect	Acid-forming
*Effect on Potassium*	Higher dietary potassium, but better intracellular shift due to carbohydrate content	Variable, generally lower K content
*Effect on Gut Microbiome*	Higher fibre → lower uremic toxins	Increases production of uremic toxins
*Impact on CKD Progression*	Associated with slower CKD progression	Higher acid load and phosphorus may accelerate CKD progression
*Association with PEW*	May reduce inflammation, supports energy intake	Higher protein quality but higher phosphorus burden

DIAAS—Digestible Indispensable Amino Acid Score; CKD—chronic kidney disease.

**Table 6:** Recommended parenteral nutrient intakes in acute kidney injury/chronic kidney disease without renal replacement therapy [27].

Energy	20–25 kcal/kg body weight/day
Amino acids	0.6–1 g/kg body weight/day *
Carbohydrates	3–5 g/kg body weight/day
Lipids	0.8–1.2 g/kg body weight/day **
Water-soluble vitamins	Normal parenteral nutrition dosage
Fat-soluble vitamins	Normal parenteral nutrition dosage
Electrolytes ***	Phosphate/potassium restriction is often necessary
Fluid ****	

* Depending on the degree of catabolism and tolerance. ** Monitoring of plasma triacylglycerides is necessary. *** Optional. **** The individual requirements vary a great deal.

## Data Availability

The original contributions presented in this study are included in this article; further inquiries can be directed to the corresponding author.

## Abbreviations

The following abbreviations are used in this manuscript:

AKI	Acute Kidney Injury
BMI	body mass index
CKD	chronic kidney disease
DIAAS	Digestible Indispensable Amino Acid Score
EA	essential amino acids
EN	enteral nutrition
ESPEN	European Society for Clinical Nutrition and Metabolism
FAO	Food and Agriculture Organization of the United Nations
IGF-1	insulin-like growth factor-1
IL-6	interleukin 6
ISRNM	International Society of Renal Nutrition and Metabolism
KDIGO	Kidney Disease: Improving Global Outcomes
KDOQI	Kidney Disease Outcomes Quality Initiative
LPD	low protein diet
MAC	metabolic acidosis
ONS	oral nutrition supplements
PEW	protein–energy wasting
PLADO	plant-dominant low-protein diets
PN	parenteral nutrition
RNS	reactive nitrogen species
ROS	reactive oxygen species
SCFA	short-chain fatty acid
TNFα	tumour-necrosis factor α
TNP	total parenteral nutrition
VLPD	very low protein diet
WHO	World Health Organization
